# 44 Endothelial Monolayers Treated with Burn Patient Plasma Exhibit Differential Gene Expression Linked to Cytoskeletal Rearrangement

**DOI:** 10.1093/jbcr/irac012.047

**Published:** 2022-03-23

**Authors:** Edward J Kelly, John W Keyloun, Bonnie C Carney, Lauren T Moffatt, Jeffrey W Shupp

**Affiliations:** Medstar Washington Hospital Center, Alexandria, Virginia; Burn Center at MedStar Washington Hospital Center, Washington DC, District of Columbia; Medstar Health Research Institute, Washington DC, District of Columbia; Burn Center at MedStar Washington Hospital Center, Washington DC, District of Columbia; MedStar Washington Hospital Center, Washington DC, District of Columbia

## Abstract

**Introduction:**

Burn shock is one of the most serious and complex complications suffered by patients following thermal injury. Endothelial dysfunction may play a role in the pathogenesis of burn shock. However, the mechanisms underlying the contribution to pathophysiology are still largely unknown. Previous studies have shown a connection between the rearrangement of cytoskeletal elements leading to increased vascular permeability. The aim of this study was to examine the differential expression of genes involved in cytoskeletal arrangement in endothelial cell monolayers treated with plasma from burn patients.

**Methods:**

Human umbilical vein endothelial cells (HUVECs) were seeded into transwell plates to form confluent monolayers. Plasma was collected from burn patients 4 hours post-admission. HUVEC cells were exposed to 10% multi-donor pooled healthy human plasma (HHP) or burn patient plasma. Monolayers were subsequently incubated with FIT-C Dextran (40,000 kD) for 2 hours. Monolayer permeability was measured with indices calculated by normalizing values to blank wells (transwell inserts) and HHP-treated monolayer FIT-C diffusion. RNA was isolated from these same cells that had increased monolayer permeability and PCR analysis was carried out using an 84 gene array of human cytoskeletal regulators (Qiagen). A Ct value of 35 was used to indicate expression and a fold change of 1.5 to indicate differential expression in the control vs. injured groups.

**Results:**

Four burn patient plasma samples were utilized to create injuries. Patients were mostly male (75%) with a mean age of 50±20 years and mean %TBSA burn of 37±34%. Differential gene expression in burn vs. HHP was compared. Ten genes showed significant upregulation (ARHGDIB, AURKA, AURKB, CCNB2, CIT, IQGAP2, VASP, SSH2, MYLK and DIAPH1). Four genes showed significant downregulation in (FSCN2, ARHGAP6, CYFIP2, CCNA1). Monolayer permeability indices showed statistically significant increases when compared to controls ranging from 3-13.33% (p < .05).

**Conclusions:**

The interplay of burn shock and endothelial dysfunction remains a complex process of which much is unknown. However, RNA analyses of burn patient plasma reveals involvement of multiple cytoskeletal regulators. Furthermore, all these samples show a concurrent increase in permeability indices when compared to controls, further strengthening the association between cytoskeletal rearrangement and endotheliopathy. Future research to better understand the specifics of these pathways could help aid in the development of more targeted treatments of endotheliopathy and burn shock. Cytoskeletal rearrangement may be an interesting target for future work to understand this mechanistic interplay.

